# Supplementary material for: The Protective Effects of Lactoferrin on Aflatoxin M1-Induced Compromised Intestinal Integrity
Source: Int J Mol Sci. 2021 Dec 28;23(1):289. doi: 10.3390/ijms23010289 (PMC8745159; doi:10.3390/ijms23010289)
Supplement: Supplementary file 1 [file ijms-23-00289-s001.zip › ijms-1483538-supplementary.pdf]

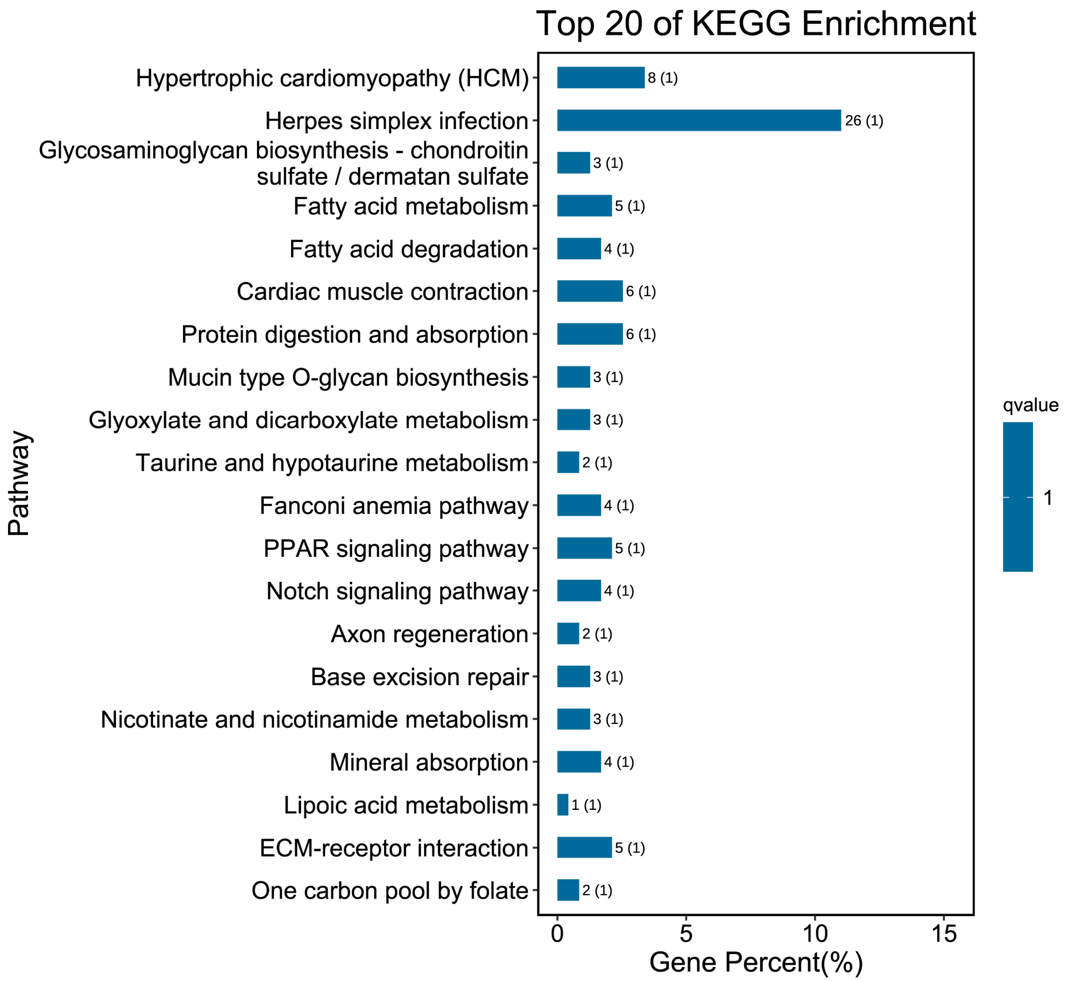

Fig.S1 The top 20 KEGG pathways enriched by the 562 unique DEGs in the combined group of LF and AFM1.

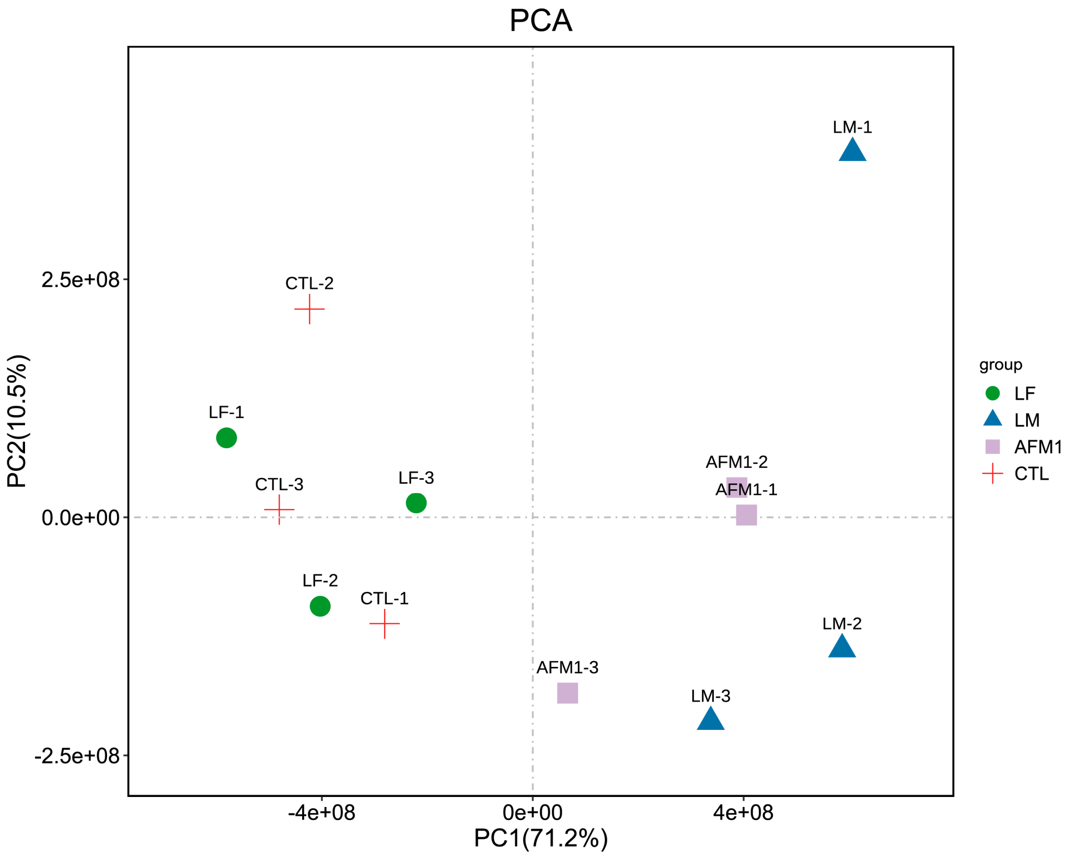

Fig.S2 Unsupervised PCA analysis for the proteome data induced by individual and combined LF and AFM1. LF represents individual 100 μg/mL LF, AFM1 represents individual 8 μg/mL AFM1, LM represents the combination of 100 μg/mL LF and 8 μg/mL AFM1.

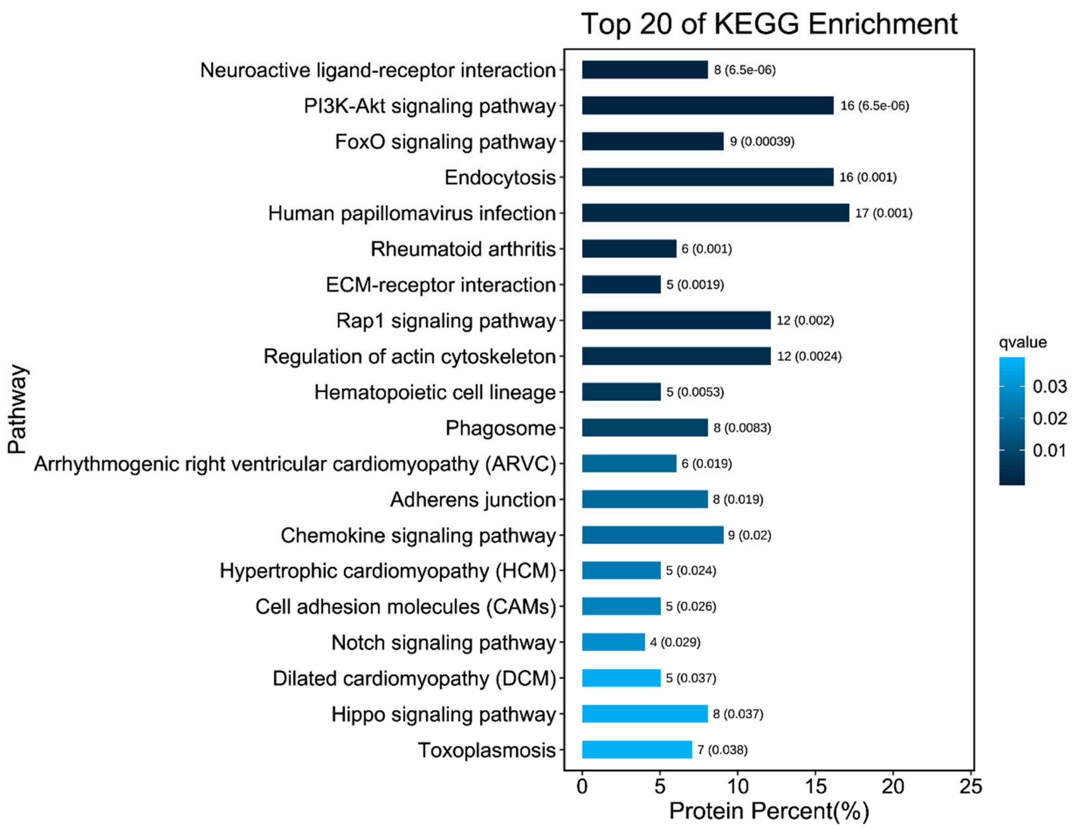

Fig.S3 The top 20 KEGG pathways enriched by the 562 unique DEPs in the combined group of LF and AFM1.

Table S1 Primers used for qRT-PCR analysis

Gene Name	Primer sequences (5'-3')
OCLN	F: 5' GGGCATTGCTCATCCTGAAG 3'<br>R: 5' GCCTGTAAGGAGGTGGACTT 3'
CLU	F: 5' GGATGCCCTAAATGAGACCA 3'<br>R: 5' TCAGGCAGGGCTTACACTCT 3'
LAMP2	F: 5' AATGCCACTTGCCTTTATGC 3'<br>R: 5' ATCATCCCCACAAATGCTTC 3'
RPS8	F: 5' CCTACCACAAGAAGCGGAAG 3'<br>R: 5' CAGGGCACGGTATTTCTTGT 3'
CLDN4	F: 5' GTCTGCCTGCATCTCCTCTGT 3'<br>R: 5' CCTCTAAACCCGTCCATCCA 3'
CLDN3	F: 5' CTGCTCTGCTGCTCGTGTC 3'<br>R: 5' CGTAGTCCTTGCGGTCGTAG 3'
JUND	F: 5' ACCCTTCTACGGCGATGAG 3'<br>R: 5' GCGTCCTTCTTCATCATGC 3'
GADPH	F: 5' GAGATTACTGCCCTGGCTCCTA 3'<br>R: 5' ACTCATCGTACTCCTGCTTGCTG 3'
